# Analysis of Promyelocytic Leukemia in Human Embryonic Carcinoma Stem Cells During Retinoic Acid-Induced Neural Differentiation

**DOI:** 10.15171/ijb.1358

**Published:** 2016-09

**Authors:** Khadijeh Karbalaie, Sadeq Vallian, Liana Lachinani, Somayeh Tanhaei, Hossein Baharvand, Mohammad Hossein Nasr-Esfahani

**Affiliations:** ^1^Division of Genetics, Department of Biology, Faculty of Science, University of Isfahan, Isfahan, Iran; ^2^Department of Cellular Biotechnology, Cell Science Research Center, Royan Institute for Biotechnology, ACECR, Isfahan, Iran; ^3^Department of Cell and Molecular Biology, Cell Science Research Center, Royan Institute for Biotechnology, ACECR, Isfahan, Iran; ^4^Department of Molecular Genetics , Cell Science Research Center, Royan Institute for Biotechnology, ACECR, Isfahan, Iran; ^5^Department of Stem Cells and Developmental Biology, Cell Science Research Center, Royan Institute for Stem Cell Biology and Technology, ACECR, Tehran, Iran; ^6^Department of Developmental Biology, University of Science and Culture, Tehran, Iran

**Keywords:** Pluripotent stem cells, Promyelocytic Leukemia, Retinoic acid

## Abstract

**Background:**

Promyelocytic leukemia protein (*PML*) is a tumor suppressor protein that is involved in myeloid cell differentiation in response to retinoic acid (RA). In addition, RA acts as a natural morphogen in neural development.

**Objectives:**

This study aimed to examine *PML* gene expression in different stages of in vitro neural differentiation of NT2 cells, and to investigate the possible role of *PML* in pluripotency and/or neural development.

**Materials and Methods:**

RA was used as a neural inducer for in vitro neural differentiation of NT2 cells. During this process *PML* mRNA and protein levels were assessed by quantitative real time RT-PCR (QRT-PCR) and Immunoblotting, respectively. Furthermore bisulfite sequencing PCR (BSP) was used to assess *PML* promoter methylation in NT2 cells and NT2 derived neuronal precursor cells (NT2.NPCs).

**Results:**

QRT-PCR results showed that, *PML* had maximum expression with significant differences in NT2 derived neuronal precursor cells relative to NT2 cells and NT2 derived neural cells (NT2.NCs). Numerous isoforms of *PML* with different intensities appeared in immunoblots of pluripotent NT2 cells, NT2.NPCs, and NT2.NCs. Furthermore, the methylation of the *PML* promoter in NT2.NCs was 2.6 percent lower than NT2 cell.

**Conclusions:**

The observed differences in *PML* expression in different cellular stages possibly could be attributed to the fact that *PML* in each developmental state might be involved in different cell signaling machinery and different functions. The appearance of different *PML* isoforms with more intensity in neural progenitor cells; may suggest apossible role for this protein in neural development.

## 1. Background

Human *PML* gene is located on chromosome 15(15q) (NC_ 000015.10; 74287014…74340160). PML‏ protein has several motifs, including an RBCC domain‏ (two zinc finger domains, B boxes, and an α-helical‏ coiled-coil motif), nuclear localization signal (NLS),‏ and a serin/prolin rich domain (1). This protein was‏ known as the one of the major components of the‏ promyelocytic leukemia nuclear bodies (PML-NBs).‏ Retinoic acid (RA) acts as a natural morphogen in neural development and as a key component of RAdependent‏ myeloid differentiation (2-4). Different‏ pluripotent cell lines were differentiated to mature *in vitro* neural cells after RA treatment.

CpG islands (or CG islands) are at least 200‏ nucleotide regions with more than 50% GC and 65%‏ CpG ratio (5,6). These islands could be considered as‏ indicative of vertebrate genome, which are present in‏ almost 70% of vertebrate promoters (7). More than‏ 50% of human genes have these islands at their promoters (8). It has been documented that housekeeping‏ genes, developmental regulatory genes, and tissue specific‏ genes have promoters that possess CpG islands‏ which are called CpG promoters (9-12). A number of‏ factors are known to contribute in the control of gene‏ expression in CpG promoters. These factors include‏ the binding of specific transcription factors such as‏ specific protein one (SP1), the presence of active epigenetic‏ marks and open chromatin structures accessible‏ for transcription machinery (13,14). Among the‏ epigenetic markers, DNA methylation has an important‏ role in the control of gene expression in CpG promoters.‏ Conversion of cytosine to methyl-cytosine in‏ CpG dinucleotides changes DNA sequence accessibility‏ for active transcription factors, producing compact‏ chromatin structures ‏ unsuitable for transcription. DNA‏ methylation as a long term epigenetic mark occurs in‏ CpG promoters of pluripotent and pluripotent associated‏ genes in stem cells (15).

## 2. Objectives

With regard to the fact that stem cells are a suitable‏ model for *in vitro* mammalian developmental studies,‏ this study aims to investigate the possible role of *PML* in pluripotency and/or neural development after RA‏ treatment. Furthermore, considering DNA methylation‏ as a key factor in the normal development and differentiation‏ process, the DNA methylation pattern of the‏ *PML* promoter in pluripotent stem cells and ECderived‏ neural cells (NCs) will also be addressed.‏

## 3. Materials and Methods

### 3.1. Culture and Differentiation of Embryonic Carcinoma Stem Cells (ECSCs)

The embryonic Carcinoma stem cell line‏ (ECSCs;NT2) was cultured in DMEM (Gibco, UK)‏ supplemented with FCS (Gibco) and 2 mM L-glutamine.‏ Cell passage was carried out by treatment with‏ trypsin (Gibco), and cells were seeded in new dishes at‏ a 1:5 ratio. Neural induction of these cells was accomplished‏ in growth medium supplemented with different‏ inducers and factors over a three-month period.‏ Initially, 2×10^4^ cells/cm^2^ were seeded in adherent tissue‏ culture dishes in the presence of 10 μM RA for a‏ month. The resultant compact neuro epithelial cells‏ were dissociated by trypsin and seeded in a 3:7 ratio in‏ new adherent culture dishes in the presence of 1 μM‏ cytosine arabinosin (Sigma, USA) only for the first‏ week of this period, 10 μM fluorodeoxyuridine‏ (Sigma, USA) and 10 μM uridine for a month. For‏ neural maturation, the apparent cell aggregates were‏ mechanically dissociated by hitting to the side of the‏ tissue culture dish. Dislodged aggregates were seeded‏ in poly-D-lysine (Sigma)-coated dishes in the presence‏ of 1 μM cytosine ara binosin (for the first week) and‏ 10 μM fluorodeoxyuridine (16).

### 3.2. RNA Isolation and Quantitative Real-time PCR

Total RNA was extracted from three different‏ stages of the neural differentiation process of NT2‏ cells by TRI reagent (Sigma, USA). Samples were‏ treated with DNaseI (Fermentas, Germany) to remove‏ contaminating genomic DNA. cDNA synthesis was‏ carried out using the Revert Aid First Strand cDNA‏ Synthesis Kit and random hexamer primers‏ (ThermoScientific). Real time PCR was performed‏ with an Applied Biosystems (version 2.1) thermal‏ cycler. Each PCR reaction was performed in triplicate‏ using SYBR Green PCR Master Mix (Takara Bio. Inc.,‏ Japan), 2.5 pm of each primer and 25 ng cDNA in a‏ final volume of 10 μL. Real time PCR data were normalized‏ by GAPDH and relative gene expressions‏ were analyzed by the comparative Ct method, 2-ΔΔCt‏ (17). (Table 1) lists the sequence and the corresponding‏ information for the Real-time PCR primers. The‏ experiments were performed three times in three separate‏ cultures in which cells were differentiated from‏ stem cell stages to mature neural cells. Data from three‏ independent repeats were assessed by one-way‏ ANOVA analysis and presented as mean ± SEM.

**Table 1:** Primers that were used for gene expression analysis by QRT-PCR

**Genes**	**Forward Primer (5'-3')**	**Reverse Primer (5'-3')**	AT^*^	**Accession No**
				
*GAPDH*	CCACTCCTCCACCTTTGACG	CCACCACCCTGTTGCTGTAG	56° C	NM_002046.3
*POU5F1*	TCTATTTGGGAAGGTATTCAGC	ATTGTTGTCAGCTTCCTCCA	60° C	NM_001173531.1
*NANOG*	CAGCTACAAACAGGTGAAGAC	TGGTGGTAGGAAGAGTAAAGG	56° C	NM_024865.2
*NESTIN*	TCCAGGAACGGAAAATCAAG	TTCTCTTGTCCCGCAGACTT	55° C	NM_006617.1
*PAX6*	CAGCTCGGTGGTGTCTTTG	AGTCGCTACTCTCGGTTTA	57° C	NM_001127612.1
*TUJ1*	AAGCCAGCAGTGTCTAAACCC	GGGAGGACGAGGCCATAAATAC	60° C	NM_006086.2
*NCAM*	CTCGGCCTTTGTGTTTCCAG	TGGCAGGAGATGCCAAAGAT	57° C	NM_181351.3
*PML*	ACCTCTGGTTTTCTTTGACCTCAAG	GAACTTGCTTTCCCGGTTCAC	62° C	NM_033238.2
*PML*-E2-E3	CGCAAGACCAACAACATC	GAACATCCTCGGCAGTAG	56° C	NM_033238.2

*: AT is annealing temperature

### 3.3. Immunoblot Analysis

Protein extracts were prepared from three stages of‏ neural differentiation process of NT2 cells by TRI‏ reagent (Sigma). Solubilized protein fraction of each‏ sample (30 μg) was subjected to SDS-PAGE electrophoresis‏ and transferred to a poly vinylidene fluoride‏ (PVDF) membrane. The membrane was soaked in 5%‏ (w/v) skim milk as a blocking solution, rabbit anti-PML‏ antibody (0.5 μg.mL^-1^, Santa Cruz, USA) and mouse‏ anti-ACTIN antibody (4 μg.mL^-1^, Sigma) as first antibodies followed by horseradish peroxidase (HRP)-conjugated‏ goat anti-mouse IgG (Dako, Denmark) and‏ HRP-conjugated goat anti-rabbit IgG (Santa Cruz) as‏ secondary antibodies. HRP-conjugated IgGs bound to‏ each protein band were visualized by an Amersham‏ ECL advance Western blotting detection kit (GE‏ Healthcare, Amersham Bioscience, UK).‏

### 3.4. DNA Extraction and Bisulfite Treatment

Genomic DNA of the NT2 cell line and NT2‏ derived neural cells (NT2.NCs) was extracted by the‏ DN easy® Blood and Tissue Kit (Qiagen, Germany).‏ Complete bisulfite conversion and DNA clean-up for‏ methylation analysis was performed by the EpiTect®‏ Bisulfite Kit (Qiagen, Germany).

### 3.5. Primer Design and Bisulfite Sequencing PCR

Meth Primer online software was chosen for primer‏ design (www.urogene.org/MethPrimer). The primers‏ used for amplification of PML were as: Forward,‏ TTTTGTAGTTTTGTTTTATTTTTTT and reverse, ATTA‏ ACTAAATCCCTTAAACTATCC. The annealing temperature‏ was 57ºC (Table 2). The PCR master mix was prepared‏ according to Herman *et al*. with some modifications‏ (18). Briefly, 300 ng of each forward and reverse‏ primers was used for PCR reactions with 1 μL bisulfite‏ modified DNA, 1×ammonium sulfate (AMS) buffer‏ (Cina Clone, Iran), 6 mM MgCl2 (Cinna Gen, Iran),‏ 1.25 mM dNTP (Cinna Gen, Iran), 0.6 U S-mar Taq (Cinna Gen) and 10 mM 2-mercaptoethanol (Sigma) in‏ a 25 μL reaction volume. PCR reaction was carried out‏ in an Eppendorf Master cycler gradient thermal cycler‏ with the following program: 95ºC for 10 min, 35 cycles‏ at 95ºC for 30 sec, 58ºC for 30 sec, and 72ºC for 1 min,‏ followed by a final extension at 72ºC for 10 min. PCR‏ products were subcloned into a pTZ57R/T cloning vector‏ (InsTAcloneTM PCR Cloning Kit, Fermentas,‏ Germany) according to the manufacturer’s protocol.‏ Ligated vectors were transferred into the DH5α strain of‏ *E. coli* and grown colonies were selected by PCR analysis‏ through both M13 and reverse sequencing primers.‏ For each group, plasmids from 10 positive colones were‏ extracted by Qiaprep® Spin Miniprep Kit (Qiagen,‏ Germany) and sequenced using standard M13 primers.‏ The obtained sequences were analyzed with bisulfite‏ sequencing DNA methylation analysis (BISMA) online‏ software that defines the methylation pattern of the *PML* promoter (19). Using the software, parameters like‏ lower threshold conversion rate and lower threshold‏ sequence identity were chosen at 90%. The upper‏ threshold of N-sites at the cytosine positions and the‏ upper threshold insertions/deletions were chosen by‏ 20%.

## 4. Results

### 4.1. Formation of Neural Precursor Cells (NPCs) and Neural Cells (NCs) from NT2 Cells

**Figure 1:**
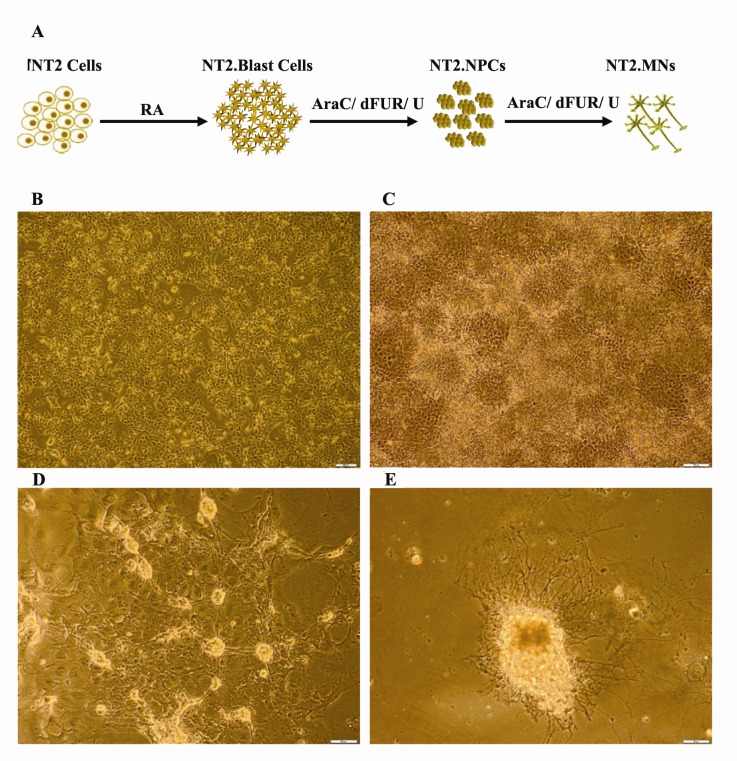
The identity of NT2 cells changed following retinoic acid (RA) treatment. A: Schematic diagram of neural induction in NT2 cells by sequential RA and mitotic inhibitor treatment. B: NT2 cell line in pluripotent state grew as cell monolayer . C: In the presence of RA, the cells form rosette-like structures contained blast cells. D: NT2.NPCs formed in the presence of mitotic inhibitors. E. Pure mature neurons (MN) appeared after three months of NT2 neural induction

NT2 cells were differentiated in three stages over a‏ three-month process (Figure 1A). Three or four angle‏ NT2 cells (3 or 4 angle) with short and fine processes‏ grew as adherent cell monolayer (Figure 1B). In culture‏ medium that contained RA, NT2 cells propagated‏ as blast slime cells such as neuroepithelial cells. As‏ shown in (Figure 1C), these blast cells formed rosettelike‏ structures in the background of NT2 cells. These‏ heterogenic cultures were treated with trypsin and recultured‏ in medium that contained mitotic inhibitors.‏ After culture, the neuronal precursor cells‏ (NT2.NPCs) appeared as shining clumps with large‏ processes in the background of non-neuronal cells‏ (Figure 1D). NT2 derived neural cells (NT2.NCs),‏ which were pure single neurons or clumps of mature‏ neurons (MN) with numerous neurites formed after‏ one ‏ month culture of NT2.NPCs in the presence of‏ mitotic inhibitors (Figure 1E).

### 4.2. Gene Expression Pattern of NT2 Cells Changes Alongside Neural Differentiation

**Figure 2:**
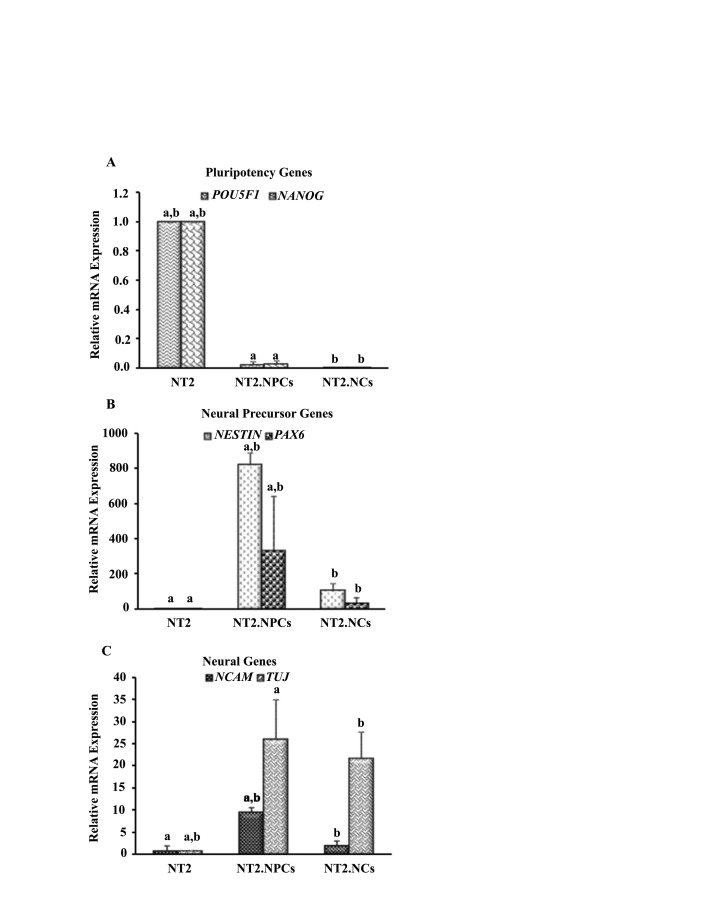
Gene expression of NT2 cells changed following retinoic acid (RA) treatment. A: Relative expression level of pluripotency genes decreased in neural cells. B: Relative expression level of neural precursor genes was higher in NT2.NPCs. C: Neural genes had maximum expression in NT2.NCs. Means with similar letters are significantly different (Tukey, LSD, p<0.05)

In NT2 cells, expressions of *OCT4* and *NANOG* decreased in NT2.NPCs and NT2.NCs (Figure 2A).‏ Expressions of *NESTIN* and *PAX6*, two neural precursor‏ markers, were significant in NT2.NPCs. However,‏ there was no significant expression of these markers in‏ NT2 cells and in NT2.NCs (Figure 2B). Expressions of‏ *TUJ1* and *NCAM*, two neuronal markers, was not‏ detected in NT2 cells (Figure 2C). In contrast,‏ increased expressions of these markers were observed‏ in NT2.NPCs and NT2.NCs. This level of expression‏ was significantly higher compared to NT2 cells‏ (Figure 2C).

**Figure 3:**
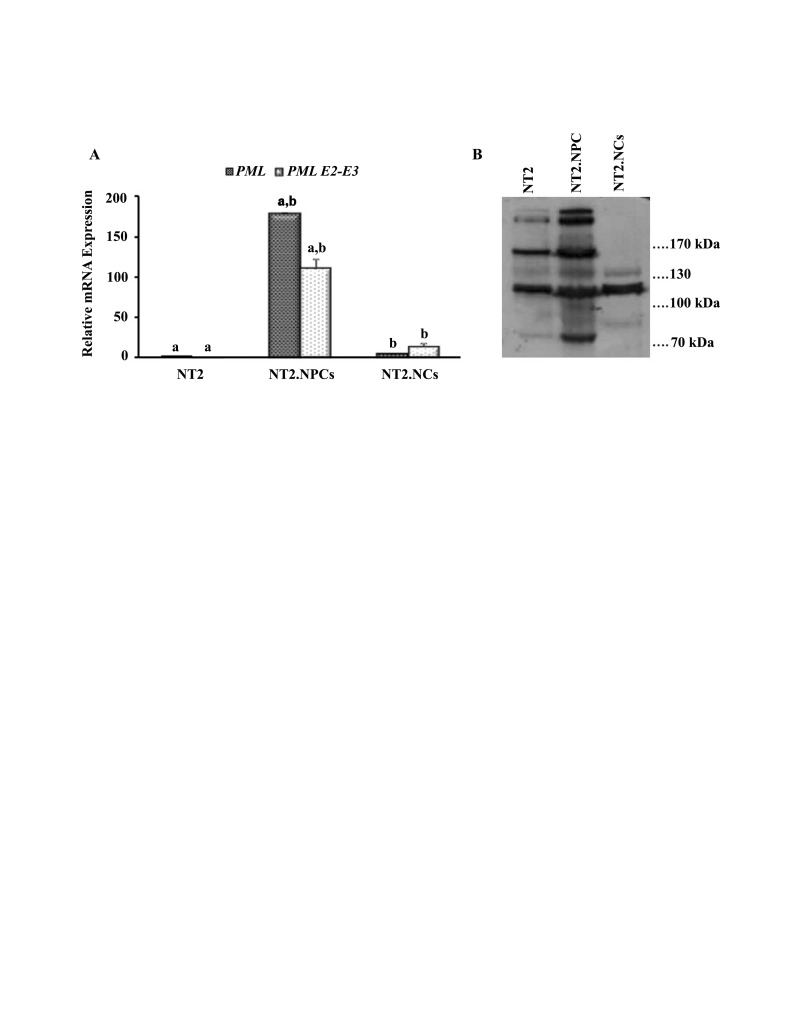
Assessment of PML expression in mRNA and protein level during NT2 cells neural differentiation. A: Relative PML expression was higher in NT2.NPCs than in NT2 and NT2.NCs. Means with similar letters are significantly different (Tukey’s, LSD, p < 0.05). B: Different PML isoforms with different intensities were detected in immunoblot analysis of PML in NT2 cells, NT2.NPCs and NT2.NCs

In parallel with these gene expression assessments,‏ a very slight level of *PML* expression was noted in‏ NT2 cells (Figure 3A). Following RA treatment and‏ neural induction, higher levels of *PML* and *PML* E2-‏ E3 expressions were detected in NT2.NPCs compared‏ to NT2 cells. However and at the end of the differentiation‏ process, *PML* and *PML* E2-E3 expressions in‏ NT2. NCs decreased with a level similar to NT2 cells‏ (Figure 3A). Immunoblotting was performed on the‏ extracts of NT2 cells, NT2.NPCs and NT2.NCs‏ showed the presence of different PML isoforms. On‏ the extracts of the NT2 cells, seven bands in the range‏ of 70-200 kDa were observed. In NT2.NPCs, the same‏ pattern with increased intensity was detected.‏ However, in NT2.NCs, there was no 170 kDa PML‏ isoform. Two sharp bands within 130 kDa were‏ observed and also the intensity of the other bands with‏ lower molecular weights was attenuated (Figure 3B).

### 4.3. Bisulfite Sequencing PCR of the PML Promoter in NT2 and NT2.NPCs

**Figure 4:**
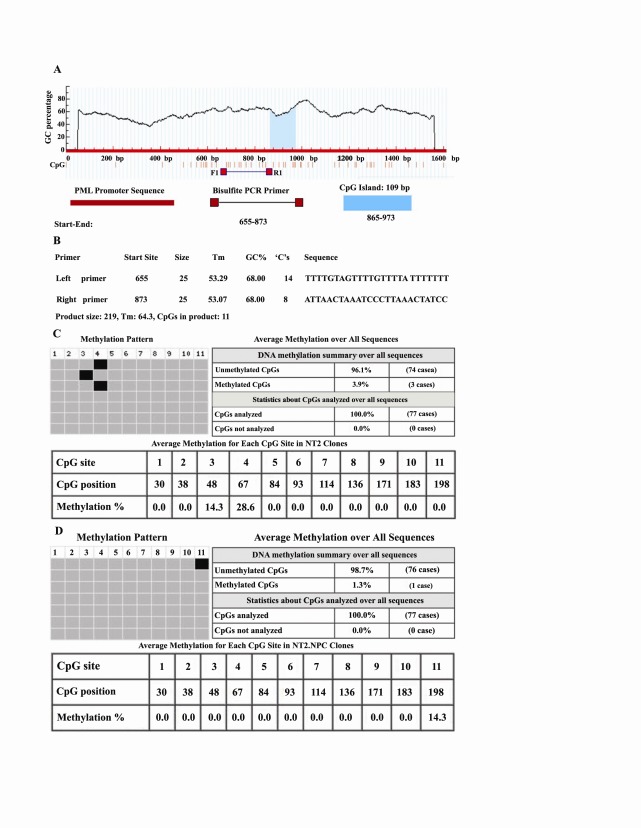
Analysis of PML promoter by Methprimer and BISMA online software programs. A: In a shematic representation of Methprimer software, the position of the CpG island with 109 base pair and the position of suitable BSP specific primer pair in upstream region of the CpG island is shown. B: Characteristics included the BSP specific primer sequences, melting temperatures and product length with the number of CpG nucleotides has been shown for the proposed PCR product. C: Results of BISMA online software analysis of *PML* promoter in NT2 cells showed one methylated CpG in three clones. D: Results of BISMA online software in NT2.NPCs showed one methylated CpG in only one clone

The human *PML* promoter region from -809/+800‏ relative to the transcription start site (74286205-74287814) was imported as an input sequence in Meth‏ Primer software (20). The promoter sequence was submitted‏ in the main page of the software and primers‏ were designed for BSP. Bioinformatic analysis showed‏ one CpG island with 109 bp at 865-973 region in a‏ TATA-less *PML* promoter (Figure 4A). This software,‏ introduced a pair of primer suitable for BSP in‏ upstream of the CpG island (Figure 3A and 4B). DNA‏ samples extracted from NT2 and NT2.NPCs were subjected‏ to BSP. PCR products were subcloned in a‏ pTZ57R/T cloning vector and final positive clones‏ used as the template for sequencing. Sequencing‏ analysis by BISMA online software showed differences‏ in NT2 cells and NT2.NPCs on the average‏ methylation for each CpG site, the number of CpG‏ sites, percent of DNA methylation, the average methylation‏ over all sequences and the average methylation‏ for each clone (Figure 4C and D). In NT2 derived‏ clones, 4 clones out of 10 clones were methylated.‏ These clones had only one methylated CpG in the third‏ or fourth CpG dinucleotide of the predicted CpG island. Methylated CpGs were located in the 48 and 67‏ region of the CpG island and methylated 14.3 and 28.6‏ percent, respectively (Figure 4C). In NT2.NPCs‏ derived clones only one CpG in the 198 region of the‏ predicted CpG island, with 14.3 percent, was methylated‏ (Figure 4D).

## 5. Discussion

The role of *PML* in granulocyte differentiation has‏ been previously reported (21). However, to date, there‏ is no report on the role of *PML* protein and methylation‏ of its gene promoter in nervous system development‏ and differentiation in human (22). Therefore, to‏ understand the eventual role of this protein in neural‏ development,NT2 pluripotent cell line were treated‏ with RA as an accepted model for *in vitro* neural differentiation‏ (3,23). After RA treatment of pluripotent‏ stem cells, NPCs with different patterns and morphologies‏ were observed. Following adherent culture‏ of NPCs, at the end of the neural differentiation‏ process, NPCs were potentiated and changed to MNs‏ with neuronal processes. It has been proved that in‏ pluripotent stem cell lines parallel to neural differentiation‏ process, expression of pluripotency genes such‏ as *OCT4* and *NANOG* decreased in stem cells and the‏ expression of neural genes including *MAP2* and *TUJ1* increased in mature neuronal cells (24-28). This phenomen,‏ similar to other pluripotent stem cell lines, was‏ observed in NT2 and NT2 derived neural cells and‏ confirmed the proposed neural differentiation process.‏ Assessment of *PML* expression in different stages of‏ neural differentiation showed that *PML* had a minimum‏ of expression in pluripotent NT2 cells and‏ reached maximum expression after RA treatment in‏ the neural precursor cells. In embryonic carcinoma‏ cells, PML-NBs were shown to be involved in OCT4‏ expression and high level of *PML* expression was‏ expected in NT2 cells. While higher *PML* expression‏ in invasive and proliferative cells of liver carcinoma in‏ contrast to lower *PML* expression in proliferative cells‏ of lung cancer has been reported (29). Therefore, in the‏ present study, since NT2 cells have been derived from‏ metastatic lung cancer, low *PML* expression in NT2‏ cells might explain their metastatic nature of origin‏ (lung) (30).

Furthermore, it is important to note that although‏ NT2 cells are pluripotent cells, they have the ability to‏ generate all three cell types of the central nervous system.‏ Therefore, they are considered as neural progenitor‏ cells (30). In view of the presence of *PML* expression in‏ proliferating neural progenitor cells of the developing‏ mouse neocortex, it is belived that *PML* probably‏ involve in the proliferation of neural progenitor cells‏ (31). In these cells, *PML* controls protein phosphatasemediated‏ dephosphorylation of retinoblastoma tumor‏ suppressor protein (32).

In human cells, alternative splicing in the carboxyterminal‏ domains of *PML* gene has generated varoiuse‏ *PML* isoforms. Regarding to this subject, assessment of‏ the relative mRNA expression level of the first and‏ longest isoform carried out with one primer pair specific‏ to the 8a-9 exon junction. Since there is no specific‏ exon junction in other isoforms (there is a similar‏ sequence), evaluation of all *PML* isoforms specifically‏ is impossible. Therefore, one primer pair ‏ complementary‏ to the second and third exon junction, called *PML* E2-E3, was used for assessment of the relative mRNA‏ expression level of other *PML* isoforms (33). The‏ results indicated that the relative mRNA expression of‏ the *PMLI* and all other *PML* isoforms during neural differentiation‏ in NT2 cells was similar in the three studied‏ stages. This could be explained by the fact that since different‏ isoforms have a different localization and probably‏ different function in transfected fibroblast cells, the‏ observed similar expression level could be possible and‏ could not reject specific function for each isoform. This‏ may indicate that all *PML* isoforms may cooperatively‏ involve in different cellular activity.‏

Our immunoblot showed several bands representative‏ of different *PML* isoforms in different stages of‏ NT2 neural differentiation process. Interestingly, similar‏ to QRT-PCR results (see above),the number and‏ intensity of bands that express the presence of different‏ *PML* isoforms were higher in NT2.NPCs. It has‏ been reported that post-translational modifications like‏ sentrinization result in different *PML* migrating isoforms‏ (34,35). Therefore these bands may relate to‏ different *PML* isoforms and or different forms of one‏ isoforms of *PML*. There is no report on the expression‏ pattern of *PML* isoforms in stem cells, nor in NCs‏ derived from *in vivo* or *in vitro* embryos or cultures. In‏ one ‏ report from Hsu and Everett only the increment of‏ *PML* and its presumed SUMO-1 modified form after‏ RA treatment has been reported in neuron-like cells‏ obtained from NT2 cells (36). It has been shown that‏ *PML* in neocortex progenitor cells controls neural differentiation;‏ and in ‏ cortex neurons by Glu1A inhibition‏ regulates synaptic strength (37). Therefore, the‏ presence of several protein bands in both pluripotent‏ cells and NCs may suggest that this protein, in addition‏ to its possible role in pluripotency and proliferation,‏ regulates synapse plasticity that has previously been‏ reported in cortex neurons. Obviously, in view of different post-translational modifications of the *PML* protein‏ in different situations (38), further studies are‏ required to fully decipher the nature of the *PML* protein‏ bands and determine which is active in pluripotency‏ and/or neural development.

‏ Despite the presence of extensive reports on the‏ different aspects of *PML* function, the importance of‏ the methylation pattern of the *PML* promoter is‏ unclear. Therefore, in the next step, the methylation‏ pattern of the *PML* gene promoter was investigated in‏ NT2 and mature neuronal cells. The process of neural‏ differentiation in NT2 cells usually lasts three months‏ and results in pure neuron cells. However, due to the‏ long term culture of final neurons, it was technically‏ impossible to prepare suitable bisulfite treated DNA‏ for bisulfite sequencing of NT2-NCs. Therefore, the‏ methylation pattern of the *PML* promoter in NT2 cells‏ as pluripotent stem cell sand NT2-NPCs as neural cells‏ examined. Our results showed that in both NT2 cells‏ and NT2-NPCs, the *PML* promoter was differentially‏ methylated. The methylation pattern of the *PML* promoter‏ differed in NT2 cells and NT2-NPCs, but in‏ both cell types the *PML* promoter showed a low degree‏ of methylation (3.9 pecent in NT2 cells and 1.3 percent‏ in NT2.NCs). Since NT2-NPCs and NT2-NCs were‏ from a neural lineage, therefore, it was expected that‏ these two cell types would have similar methylation‏ patterns. The observed differential methylation pattern‏ could be discussed in relation to the expression pattern‏ of *PML* in these cells.

As mentioned above, quantitative RT-PCR (QRT-PCR) results showed that relative *PML* expression in NT2.NPCs was 30-fold more than NT2 cells. However according to our data, it is believed that the DNA methylation pattern could not solely describe the observed difference. It is possible that other factors may have contributed in this phenomenon. Further studies are required to determine if the difference in *PML* expression during neural differentiation of NT2 cells is related to differential CpG methylation or whether other factors regulate *PML* expression.

## Acknowledgements

This study was funded by a grant provided from‏ Royan Institute.
